# A multilayered approach of Si/SiO to promote carrier transport in electroluminescence of Si nanocrystals

**DOI:** 10.1186/1556-276X-7-200

**Published:** 2012-03-27

**Authors:** Ding Li, Yong-Bin Chen, Yong Ren, Jiang Zhu, You-Yuan Zhao, Ming Lu

**Affiliations:** 1 Department of Optical Science and Engineering, and Shanghai Ultra-Precision Optical Manufacturing Engineering Center, Fudan University, Shanghai, 200433, China

## Abstract

The electroluminescence (EL) and photoluminescence of Si nanocrystals (Si-nc) from multilayered samples of Si/SiO are investigated. Si-nc are formed within Si and SiO layers after furnace annealing. It is found that the presence of Si interlayers creates extra carrier paths for EL emission. A comparative study is further performed on a multilayered Si/SiO sample and a single-layered one with Si and SiO homogeneously mixed. Both samples have the same ratio of Si to O and the same contents of Si and O. The multilayered sample is found to have higher EL intensity, less turn-on voltage, lower resistance, and higher current efficiency than the single-layered one. The results indicate that Si interlayers in Si/SiO may act as carrier channels, which promote carrier transport and enhance the EL emission of Si-nc.

## Background

Development of highly luminescent Si is of vital importance for Si optoelectronics [1]. At present, Si nanocrystals (Si-nc) embedded in SiO_2_, or SiO_2_:Si-nc, have been widely accepted as a promising material for Si light emission due to their stable light emission, robust structure, and stimulated emission feature (JZ and ML, unpublished data) [1-26]. Researchers have been focused on the enhancement of photoluminescence (PL) of Si from the SiO_2_:Si-nc [3,6,7,11,20,24] and that of electroluminescence (EL) of Si [14-19,21,25]. One of the key problems regarding the enhancement of EL emission is how to promote the carrier (electron or hole) transport [21,25,26]. The Si_3_N_4 _:Si-nc has once been adopted since the bandgap width of Si_3_N_4 _is less than that of SiO_2_, so carrier transport within Si_3_N_4 _would become easier than that within SiO_2 _[25]. For the same reason, lateral carrier injection has been suggested recently for the sample of SiO_2_:Si-nc/SiO_2 _[21]. However, a matrix with a small bandgap width does not favor carrier confinement within Si-nc, which tends to degrade light emission of Si-nc. For instance, it is found that the PL intensity of Si-nc from amorphous Si:Si-nc/SiO_2 _is one order of magnitude less than that of SiO_2_:Si-nc/SiO_2 _(JZ and ML, unpublished data). In this work, we propose another approach to improve carrier transport, that is, an approach of multilayered structure of Si/SiO. After thermal annealing, Si-nc are formed in both Si and SiO layers. Through the investigation of PL and EL emissions from multilayered Si/SiO, it is found that Si interlayers help to facilitate carrier transport. Further comparative studies on a multilayered Si/SiO sample and a single-layered one, with Si and SiO homogeneously mixed, indicate that Si interlayers in Si/SiO act as carrier channels that promote carrier transport and enhance the EL emission of Si-nc.

## Methods

### Preparation of samples

Forty-layered Si/SiO samples were prepared by evaporating Si and SiO alternatively by electron-beam and resistance heating, respectively, onto *p*^+^-type Si substrates (0.5 to approximately 1 Ω·cm) in a vacuum chamber with a base pressure less than 5 × 10^-5 ^Pa. An *ex-situ *furnace annealing in nitrogen atmosphere at 1,100°C was performed for 1 h to induce phase separation (JZ and ML, unpublished data) [3,4,7,11] to form Si-nc. The thickness of SiO remained at 3.75 nm, and that of Si interlayer (*d*_Si_) was variable.

For a comparative study, we chose a 40-layered Si/SiO sample with thicknesses of SiO and Si layers being 3.75 and 1.00 nm, respectively, and a single-layered sample prepared by co-evaporating Si and SiO with the same ratio of Si to O and the same contents of Si and O as those of the multilayered sample. For further EL identification, a pure SiO_2 _sample with a thickness of 150 nm was prepared by electron beam evaporation, followed by furnace annealing at 1,100°C in nitrogen for 1 h.

For EL emission, Al was firstly evaporated on the backside of the *p*^+^-type Si substrate of the SiO_2_:Si-nc; then, the sample was annealed *ex situ *in nitrogen at 480°C for 10 min. A ring of Al electrode was finally evaporated onto the top surface of sample.

### Monitoring, characterization and measurements of samples

Film thickness was monitored by a calibrated microbalance (MDC-360, Maxtek Digicom Limited, Shenzen, China). The PL spectra were recorded on a fluorescence spectrometer (F-4500, Hitachi High-Tech, Minato-ku, Tokyo, Japan) with a xenon lamp as an excitation source. The wavelength of the selected exciting beam was 300 nm. Forward-biased voltage was applied to the EL device. EL emission was also recorded with the spectrometer (Hitachi, F-4500). The biased voltage as well as voltage and current readings were provided by a source meter (2400, Kiethley Instruments Inc., Cleveland, OH, USA). High-resolution transmission electron microscopy (HRTEM) was measured on an electron microscope (G2 F20, Tecnai, Amsterdam, The Netherlands) to examine the formation of Si-nc. Reflectance spectra were measured with a spectrophotometer (UV-3101 PC, Shimadzu Corporation, Nakagyo-ku, Kyoto, Japan). *In this study, all the samples refer to those that have been furnace-annealed at 1,100°C for 1 h in nitrogen*.

## Results and discussion

**Figure 1:**
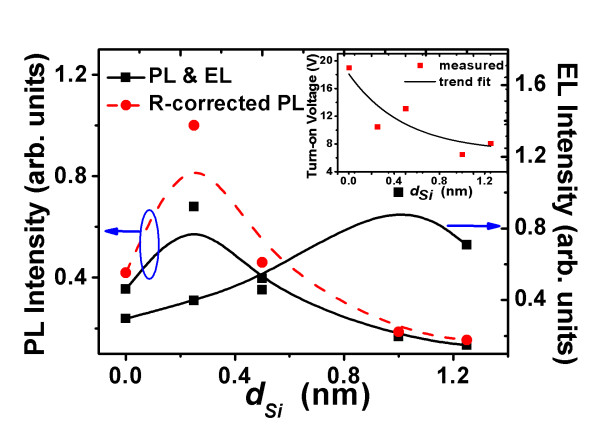
**The PL and EL intensities of Si/SiO samples versus thickness of Si interlayer, *d*_Si_**. The dashed curves are for PL intensities after *R *correction. The inset gives the turn-on voltage versus *d*_Si_.

**Figure 2:**
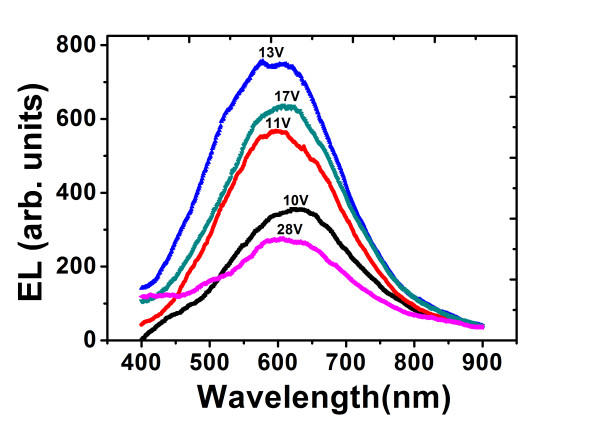
**The EL spectra with different biased voltages for one sample of Si/SiO**.

Figure 1 shows the evolutions of PL and EL intensities of Si-nc from Si/SiO as functions of *d*_Si_. In addition to the number of radiative centers, the EL intensity depends on other parameters including applied voltage, temperature change, injected current, and the number of non-radiative centers that are intrinsically present and extrinsically produced during carrier injection. The EL intensity increases at first with the increasing voltage (hence the increasing current), and after a certain voltage, the EL intensity starts to drop down due to the overwhelming effect of non-radiative centers induced by carrier injection [8,9]. Figure 2 gives an example. The shift of peak position with the voltage has been attributed to the size distribution of Si-nc [9]. The EL spectra thus selected are those that possess the highest intensities during the biased voltage ramping. The following EL intensities will all refer to this type of situation unless otherwise stated. It is seen that with increasing *d*_Si_, the PL intensity increases at first, and after reaching a maximum, it decreases. The rise of PL intensity could be attributed to the additional Si-nc formed due to the presence of Si interlayers [24,27]. With further increase in *d*_Si_, the number of 'large' Si crystallite with size greater than the Si Bohr's radius (4.3 nm) [28] increases in both Si and SiO layers due to the ripening process [29], which consumes the excess Si content but contributes little to PL emission, leading to a decrease in PL intensity. Detailed description and identification of the evolution process of PL has been presented in the study of Ren et al. [24]. A similar trend also exists for the EL evolution but with a greater optimized *d*_Si_. The inset of Figure 1 gives the turn-on voltage as a function of *d*_Si_. The trend indicates that with the increasing *d*_Si_, extra conductive percolation paths of carriers for EL are created, which promotes carrier transport. To compare the evolutions of EL and PL intensities with increasing *d*_Si _more sensibly, reflection (*R*) correction is performed for the PL intensity, that is, each PL intensity is multiplied with 1/(1 - *R*), where *R *is the measured reflectance at *λ *= 300 nm, as shown by the dashed curve in Figure 1. As compared to the PL emission, the EL intensity depends not only on the density of Si-nc, but on the carrier transport as well. The difference in the maximal PL and EL intensity in Figure 1 arises from their different excitation processes, sample geometries, and measuring conditions. The larger optimized value of *d*_Si _of EL could be due to a compromise between the decreasing density of Si-nc as reflected by the PL intensity and the increasing carrier transport as suggested by the result of the turn-on voltage in the inset of Figure 1.

**Figure 3:**
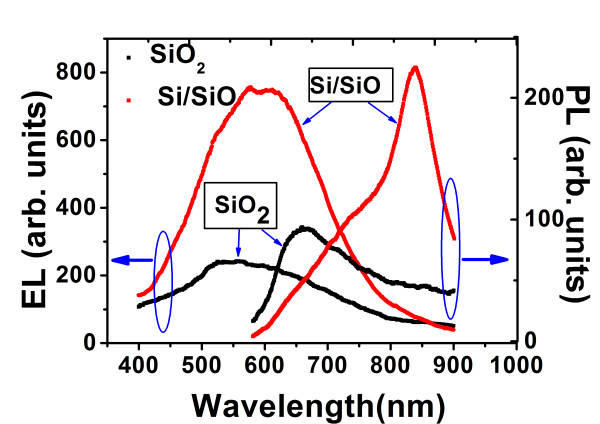
**The PL and EL spectra from one Si/SiO sample and the sample of SiO_2_**.

In the discussion of the trend of EL intensity in Figure 1, it has been implicitly assumed that the EL is from Si-nc. However, the origin of EL is complex. At present, it is generally accepted that the origin of EL is related to oxygen vacancy-related defects within SiO_2_, the interface states of Si-nc, and Si-nc themselves [8,14-21]. Figure 3 shows the PL and EL spectra of a Si/SiO sample with a *d*_Si _of 1.00 nm. With respect to the PL spectrum of Si-nc, the EL has a broad spectral width and a large blueshift, which can be ascribed to the effects of direct electron tunneling [8] and band filling [19], and various defect states. For further identification, in Figure 3, the PL and EL spectra from a pure SiO_2 _sample with a thickness of 150 nm after annealing at 1,100°C are given. The total thickness of SiO was 150 nm, too. The structure peaking at 655 nm in the PL spectrum is known from the oxygen vacancy-related defects within SiO_2 _such as non-bridging oxygen hole centers and the neutral oxygen vacancies (see the work of Lin et al. [8] and references therein). The EL of the SiO_2 _sample differs from that of the Si/SiO, namely, the overall peak has a blueshift of approximately 45 nm, and the intensity is only approximately 33% of Si/SiO. Therefore, it is concluded that the contribution from the defect in SiO_2 _to the EL of Si/SiO is of minor importance, and those from Si-nc and Si-nc-related defects are dominant in the EL emission. We notice that for SiO_2_, the EL also has a broad spectral width and blueshift with respect to the PL, indicating that effects of direct electron tunneling and band filling might be at work, too.

**Figure 4:**
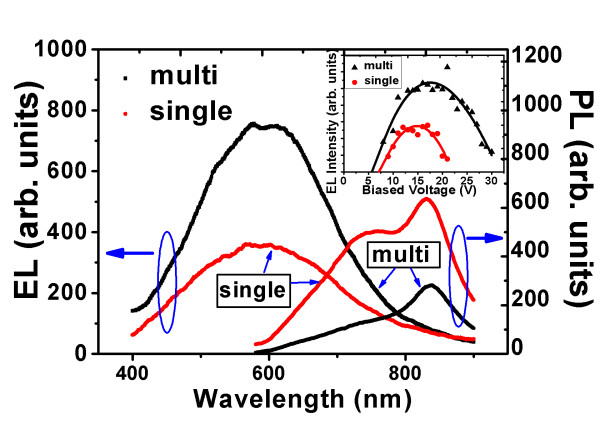
**The *R-*corrected PL and EL spectra from 'multi' and 'single'**. The inset shows the trends of EL intensity versus bias voltage for the two samples.

**Figure 5:**
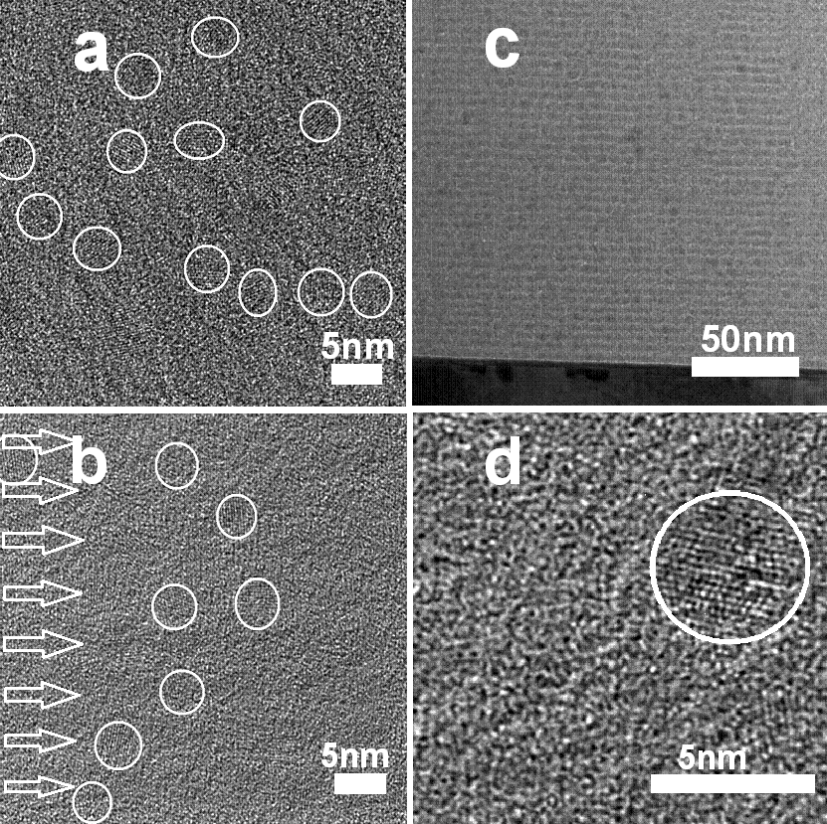
**HRTEM images for the samples**. 'Single' (**a**) and 'multi' (**b**). (**c**) The TEM of multi with low magnification, and (d) a Si-nc image.

To further explore the role played by the multilayered structure in promoting carrier transport, two samples are studied. One is a 40-layered sample of Si/SiO termed as 'multi', with thicknesses of SiO being 3.75 nm and *d*_Si _1.00 nm; the other is a single-layered one termed as 'single' with Si and SiO homogeneously mixed, followed by furnace annealing. For both samples, the ratio of Si to O and the contents of Si and O are identical. Figure 4 shows that the PL intensity from multi is only approximately 0.4 times that of single after *R *correction. Hence, the density of Si-nc is supposed to be greater in single, which is confirmed by the HRTEM results in Figure 5a, b. Two typical images for single and multi are presented here, and the densities of Si-nc with size no greater than 5.0 nm are 9.0 ± 3.4/(46 × 46 nm^2^) for single and 6.7 ± 1.6/(46 × 46 nm^2^) for multi as averaged over a dozen of HRTEM images. However, in Figure 4, the EL intensity of multi is approximately 2.1 times that of single. The current efficiency defined as the maximal EL intensity divided by the current density is 597.8 mA cm^-2 ^for multi, and 483.9 mA cm^-2 ^for single. Also, mean resistances of both samples are measured and calculated to be 84.3 ± 10.2 Ω for multi and 117.8 ± 8.7 Ω for single. Furthermore, in the inset of Figure 4, the EL intensity versus the bias voltage is given. The derived turn-on voltage for multi is 5.7 V, and that of single is 7.2 V. All these results indicate that although the two samples possess the same ratios of Si to O and the same contents of Si and O, with the number of Si-nc of single even larger than that of multi, the sample of single with excess Si randomly distributed is less efficient in carrier transport than the sample of multilayered Si/SiO. Let us compare the EL efficiency of the two samples. For multi, the input power is 15 V × 195 mA = 2.9 J; for single, it is 16 V × 150 mA = 2.4 J. The EL efficiency is defined as the EL emission energy divided by the input energy. Considering that the EL intensity of multi is 2.1 times that of single, the EL efficiency of multi is thus 2.1/(2.9 × 2.4) = 1.74 times that of single. In fact, the number of Si-nc in multi is approximately 0.4 times that of single as we estimated from the PL results of Figure 4 and the data of Figure 5; the EL efficiency of Si-nc of multi is therefore 1.74/0.4 = 4.3 times that of single.

**Figure 6:**
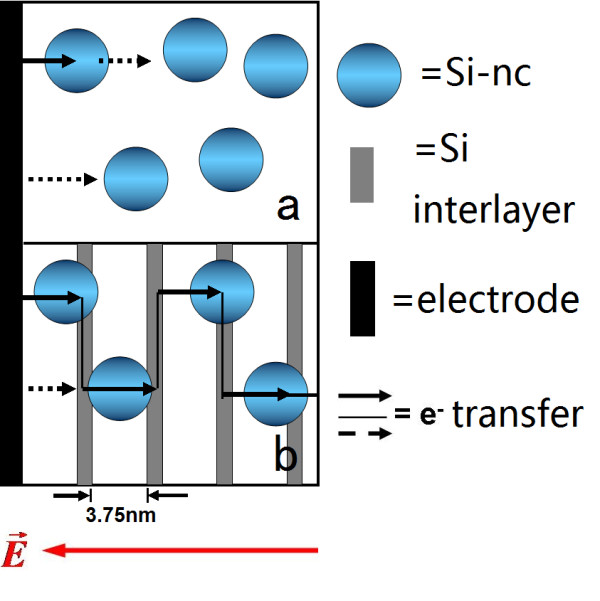
**Diagrams of carrier (electron for example) transport**. This carrier transport occurred in the 'single' (**a**), and 'multi' with Si layers as carrier channels (**b**).

It is known that Si-nc are also formed in the Si interlayers after thermal annealing [24,27], which could be clearly seen for thicker *d*_Si _[24]. In Figure 5b, the arrows indicate the positions of Si interlayer. Figure 5c gives a TEM of multi with a lower magnification, and Figure 5d shows a single Si-nc. Considering that the energy levels of various Si crystallites in Si interlayers are close and their bandgap widths are much smaller than that of SiO_2_, carriers injected into Si interlayers would move more freely within the layers than in SiO_2 _matrix. With this in mind, a scenario is supposed in Figure 6. Two diagrams of carrier (electron for instance) transport within the single and multi samples are given, respectively, under a biased electric field. Electrons injected from the electrode on the left hand side, for instance, tunnel through the SiO_2 _barriers and reside on Si-nc. On the other hand, holes are injected from the *p*^+^-type Si on the right hand side. In the single sample, further transport of electron would be hindered by sufficiently wide barriers of SiO_2 _before it reaches the next Si-nc as illustrated in Figure 6a by the dashed arrows. However, when the electron from a Si nanocrystal within SiO_2 _faces a Si interlayer, it would enter the Si layer readily and then move within the layer either 'upward' or 'downward' until it encounters a Si nanocrystal in the SiO_2 _layer and jumps onto it via direct tunneling under the bias. This process could continue until the electron meets and recombines a hole that has been injected from the opposite direction at a certain Si-nc to give off EL emission. Thus, the Si interlayers in multi act as a kind of carrier channels, and the carriers may jump from one Si-nc to another efficiently in the manner as illustrated in Figure 6b. Figure 6 explains why the efficient carrier transport in multi is more efficient than in single. In fact, in the work of Berghoff et al. [26], they also studied the charge transport within Si/SiO*_x_*, although the EL emission of Si-nc was disregarded there. They found that the charge transport is improved in Si/SiO*_x _*as compared with that in Si/SiO_2_. They attributed this improvement to the narrowing of the barrier width within the SiO*_x _*layer because of the formation of Si-nc, a model similar to that in Figure 6.

## Conclusion

We report that a multilayered approach of Si/SiO can promote carrier transport for EL emission by establishing carrier channels made of Si interlayers. To promote carrier transport along the normal of the thin film surface within SiO_2_: Si-nc, one normally tries to align Si-nc closely along the direction of the electric field so that carrier tunneling can become easier. However, how to align the self-organized Si-nc remains an open problem so far. The approach of a multilayered structure of Si/SiO proposed here might, to some degree, circumvent this problem and help to enhance the EL emission of Si-nc.

## Competing interests

The authors declare that they have no competing interests.

## Authors' contributions

DL prepared all the samples and measured and analyzed the PL and EL data. YC and YR measured the HRTEM data. JZ helped to prepare samples. YZ and ML designed the experiments, and ML wrote the manuscript. All authors read and approved the final manuscript.
